# The Role of Human Behavior in *Plasmodium knowlesi* Malaria Infection: A Systematic Review

**DOI:** 10.3390/ijerph19063675

**Published:** 2022-03-19

**Authors:** Nurul Athirah Naserrudin, Rozita Hod, Mohammad Saffree Jeffree, Kamruddin Ahmed, Richard Culleton, Mohd Rohaizat Hassan

**Affiliations:** 1 Department of Community Health, Faculty of Medicine, Universiti Kebangsaan Malaysia, Kuala Lumpur 56000, Malaysia; drathirah85@gmail.com (N.A.N.); rozita.hod@ppukm.ukm.edu.my (R.H.); 2 Sabah State Health Department, Ministry of Health, Kota Kinabalu 88590, Malaysia; 3 Borneo Medical and Health Research Centre, Faculty of Medicine and Health Sciences, Universiti Malaysia, Kota Kinabalu 88400, Malaysia; saffree@ums.edu.my (M.S.J.); ahmed@ums.edu.my (K.A.); 4 Department of Public Health Medicine, Universiti Malaysia Sabah, Kota Kinabalu 88400, Malaysia; 5 Department of Pathobiology and Medical Diagnostics, Faculty of Medicine and Health Sciences, Universiti Malaysia Sabah, Kota Kinabalu 88400, Malaysia; 6 Department of Molecular Parasitology, Proteo-Science Centre, Ehime University, Matsuyama 791-0295, Japan; culleton.richard.oe@ehime-u.ac.jp

**Keywords:** *Plasmodium knowlesi*, malaria, zoonotic disease, human behavior, prevention, intervention

## Abstract

Objectives: *Plasmodium knowlesi* is a non-human parasite that causes zoonotic disease in humans. This systematic review aims to highlight and summarize studies describing human behaviors and activities that expose humans to mosquito bites. *Design:* English entries in PubMed, Web of Science, and Science Direct from 2010 to 2020 were systematically perused, and the results were synthesized. Methodological quality was assessed using the Joanna Briggs Institute quality appraisal checklists. Setting: Studies that described malaria preventive measures were included. Laboratory, in vivo, in vitro, and animal studies were excluded. Primary and secondary outcome measures: The main outcome of the review was findings from studies describing the behavior that exposed a person or a group to *P. knowlesi* infection. Results: Twelve eligible studies were of good or medium quality. Attitude, disease misconceptions, perceived threat of disease, lack of motivation, and supernatural or traditional beliefs causing individuals to seek treatment from traditional healers influenced the exposure of individuals or communities to *P. knowlesi* malaria. Other factors were forestry activities (2.48, 1.45–4.23,95% CI, *p* = 0.0010) and sleeping outdoors (3.611, 1.48–8.85, 95% CI, *p* = 0.0049). Conclusions: Future studies must consider the importance of human behavior and community perspective on the infection to provide novel information to improve the current zoonotic malaria programs. Policymakers should concentrate on understanding human behavior and activities that expose individuals or communities to mosquito bites, in order to better design socially feasible interventions.

## 1. Introduction

*P. knowlesi* is a zoonotic disease transmitted by the *Anopheles* mosquito, which harbors the *Plasmodium* parasite [1]. Previously, the disease was believed to occur only among the *Macaca fascicularis* and *M. nemestrina* monkeys [1], found largely in southeast Asia [1]. In 2004, *P. knowlesi* infection was detected in a large section of the community in Sarawak, Malaysia [1]. Despite the success of malaria elimination programs worldwide [2,3,4], these zoonotic infections have been observed to be exponentially increasing among humans in Sabah, Malaysia [2], and nearby countries such as Indonesia [5,6], Vietnam [7,8], and Cambodia [9]. Patients experienced symptoms such as fever, myalgia, and headache, and in severe cases, kidney complications and fatalities [1,2].

The vectors of *P. knowlesi* are *Anopheles* mosquitoes of the *Leucospyrus* group [10]. There is heterogenicity in vector species across geographical regions in southeast Asia (SEA). For example, the species, *Anopheles balabacensis,* from the *Leucospyrus* complex, is widely distributed in east Malaysia, the Philippines, and Indonesia [10]. *Anopheles cracens* from the *Dirus* complex is found in Indonesia, West Malaysia, and Thailand, while *Anopheles dirus* is the primary vector in Cambodia, Laos, Thailand, Vietnam, and China [10].

The distribution of both adult mosquitoes and breeding sites provides important vector control evidence. These forest-dwelling vectors favor humid and covered forest and breed in temporary habitats such as puddles, ground holes, stream margins, and wheel tracks. The *Anopheles balabacensis* favors secondary forest areas surrounded by hilly areas, and estates such as oil palm estates and rubber plantations [11]. These environments increase the risk of spillover as the long tail macaques, *Anopheles* mosquitoes, and humans live in close interaction [11]. In Sabah, Malaysia, the breeding sites were also influenced by season. The rainy seasons from December to February and May to July provide temporary aquatic habitats for mosquito larva [10,12].

These mosquitoes are exophagic and tend to bite outdoors. The feeding behavior of *Anopheles* mosquitoes from the *Leucosphyrus* group peaks immediately after dark [10,12]. Chua et al. [12] stated that mosquito bites peak at around 18:00 to 22:00 h. Studies show that people are exposed to mosquito bites when they perform activities in the forest or return home from farms [10,11,12]. Recently, the *Umbrosus* group vector was found to bite humans earlier, around 07:00 to 11:00 h in Sarawak, Malaysia [13].

Studies have established that communities living on the edge of the forest, or performing forestry work, are at higher risk of developing a *P. knowles* infection [2,5,6,14,15,16]. Even travelers traveling to *P. knowlesi* affected regions are at risk to the *P. knowlesi* disease exposure [17]. Given the presence of asymptomatic *P. knowlesi* cases among household members and those not performing forest-related activities [2], there are gaps in the identification of other human activities or behaviors that put communities at risk.

Controlling zoonotic malaria is challenging, in part, due to uncertainty regarding vector distribution. To date, studies attempting to understand vector resting behavior have been unsuccessful due to difficulties in catching the vector in their natural habitats [10]. With the ongoing deforestation and changes in land use caused by anthropogenic activities, vector distributions are changing rapidly [10,12]. While significant attention has been given to vector behavior, to our knowledge, no comprehensive review has been conducted on human behaviors that influence exposure to mosquito bites, causing *P. knowlesi* infection. Previous studies on human malaria have described various risk factors such as non-adherence to vector control tools [18,19,20,21], beliefs, and perceptions [22,23,24,25,26]. Despite extensive planning and intervention programs, the increasing incidence of *P. knowlesi* annually is a threat to public health [2,3]. Human behavior is a critical factor that should be explored and considered to improve malaria intervention [27]. The importance of tailored approaches to malaria varies across populations and contexts [27]. Acknowledging how human behavior plays a role in malaria exposure, including exploring the drivers and barriers, could guide towards more effective strategies of human-centered design approaches [27] and sustainable malaria intervention [28,29]. Therefore, understanding human behavior for *P. knowlesi* malaria is essential in view of its complexity due to the presence of its simian reservoir. We aim to determine how human behavior contributes to *P. knowlesi* exposure towards facilitating the foundation of more effective malaria control and sustainable design of *P. knowlesi* malaria intervention.

### Strengths and Limitations of This Study 

1. This review highlights exploratory studies on human behavior affecting exposure to *P. knowlesi* malaria

2. This systematic review addresses how human behavioral factors contributed to malaria preventive measures in communities exposed to *P. knowlesi* infection

3. This study highlights the need to integrate studies on human and vector behavior for a more robust assessment of the population at risk to *P. knowlesi* malaria

4. The communities’ beliefs, drivers, and barriers influence behavior that affects exposure to *P. knowlesi* malaria

5. This review includes studies from 2010 to 2020 as Malaysia began diagnosis of human *P. knowlesi* in 2009; thus, studies prior to 2009 provide limited information.

## 2. Methods

This systematic review was conducted in accordance with the Preferred Reporting Items for Systematic Reviews and Meta-Analyses (PRISMA) guidelines for 2020 [30]. Using the Population, Intervention, Outcome (PIO) format, the following research question was explored: In a community, or for a person in *P. knowlesi* malaria affected areas (P), does human behavior expose the community or person (I) to *P. knowlesi* infection (O)?

The systematic review focuses on areas at risk of *P. knowlesi* infection, as described in previous studies [2,31]. A search was performed using three electronic databases: PubMed, Science Direct, and Web of Science (WoS). Gray literature was searched from World Health Organization (WHO) webpages and related articles were searched using Google Scholar with key terms. 

The keywords have been defined based on the objectives of the study. In this review, the term “behavior” includes a broad pattern of human activities, locations, and sleeping patterns that expose humans to malaria transmission, and refers to activities that occur in and outside the home and within the community [32,33]. The terms “behavior” and “activities” are used interchangeably here. Additionally, behavior includes anything a person does in response to internal or external events, either using motor muscle function, verbal behavior, or any physical event in the body controlled by the brain [34]. To avoid missing studies that did not directly draw on *P. knowlesi* behavior literature, but might be possibly aligned with the selection criteria, we included non-Medical Subject Headings (MeSH) of “behavior”, which are “activity” and “exposure” based on the definition in this review, and the non-MeSH term “human” to include only studies conducted on humans. We used the Boolen operators “AND” and “OR” to combine the keywords for the final search strings: (knowlesi) AND (behavior OR behaviour OR exposure OR activit*/activity OR activities) AND (human). The search term was individualized for each database with results entered in a Microsoft Excel file. The key terms (knowlesi) AND (behavior OR behaviour OR activity OR activities OR exposure) AND (human) (Supplementary Table S1). Two lead authors pilot tested the final search strings in three research databases (PubMed, Science Direct, and WoS) to identify potential problems. We searched three academic databases to ensure that the studies were peer reviewed. Three authors ran the finalized search strings in the selected research databases. Subsequently, one author reran the final search strings in all three databases for validation purposes.

After filtering for and manually excluding duplicate, non-English, and animal studies, the publications were assessed for their relevance to the inclusion criteria by screening their titles and abstracts. Full texts of studies that appeared potentially relevant went through a secondary screening. To increase the robustness and yield of the review, manual searches for additional articles were identified by backward citation screening based on the eligible articles from the respective databases. 

The review was limited to articles published between 1 January 2010 and 31 December 2020. Moreover, as the review only included articles published in English, it may have failed to include evidence published in another language, such as from Indonesia, Malaysia, and other countries. The review was registered under the PROSPERO protocol CRD42021251323. Table 1 shows the inclusion and exclusion criteria used for the studies.

### Data Extraction, Quality Assessment, Risk of Bias, Data Synthesis, and Data Checking

Data were extracted by three reviewers to reduce the risk of bias and human error, who independently reviewed the search results to identify relevant articles according to the inclusion and exclusion criteria. A data checking step was also performed in which the included article review was independently compared in an extraction sheet to detect any mistakes in the extracted data [35]. Any discrepancies were resolved through consensus, with help from the fourth, fifth, and sixth authors. All included studies were processed to determine the quality of analysis relevant to the research methodology. 

The Joanna Briggs Institute critical appraisal tools are recognized as reliable for investigating variations in study design, including observational studies, case series, case reports, and qualitative studies [36]. Supplementary Table S2 provides the quality assessment checklist for this review. Supplementary Table S3 includes details for all included papers relating to their citation, year of publication, study sample, sample size, study design, the method for collecting data on human behavior, results, behavior or activities that exposed individuals to *P. knowlesi* infection, study bias, and a quality assessment grade. 

## 3. Results

### 3.1. Description of Studies

**Figure 1:**
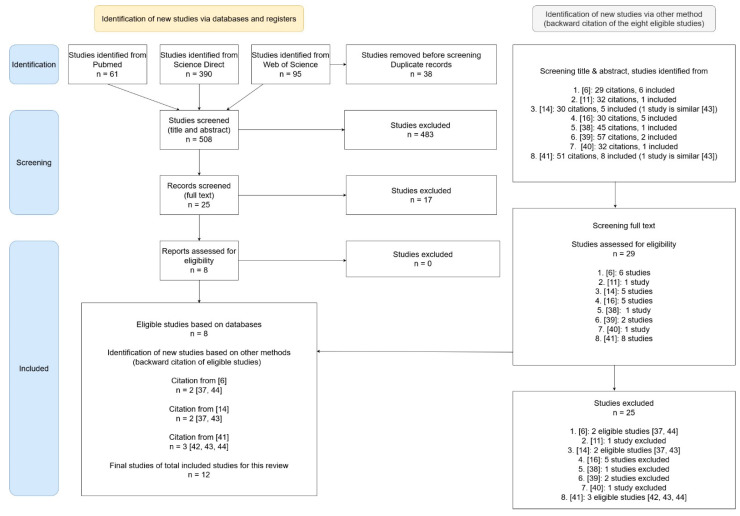
PRISMA 2020 flowchart for the literature screening process of the study [6,11,14,16,37,38,39,40,41,42,43,44].

The initial literature search returned 61 studies from PubMed, 990 from Science Direct, and 95 from Web of Science (WoS). Of the 990 articles in Science Direct only 390 were research articles and case reports, and were thus included in the screening. No related studies were found on the WHO website. After excluding duplicate, non-English, and animal studies, 508 citations remained. After screening the titles and abstracts, 25 papers appeared potentially relevant and were examined in full. Of these, eight publications were included in this study. Four more studies were included following backward citations from these eight studies, resulting in a total of 12 eligible studies (Figure 1).

**Figure 2:**
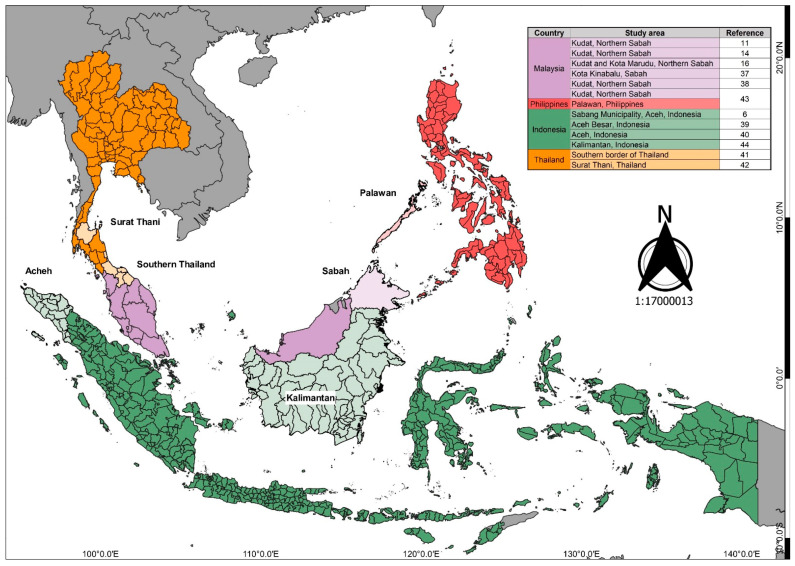
Map of study locations [6,11,14,16,37,38,39,40,41,42,43,44].

The selected studies had been conducted at the community level in several countries in the Western Pacific region: five in Malaysia [11,14,16,37,38], three in Indonesia [6,39,40], two in Thailand [41,42], and one in Malaysia and the Philippines [43]. The study by Fornace et al. [43] was undertaken in the northern part of Sabah, Malaysia, as well as the southern part of the Philippines, which have relatively homogenous populations with minimal variation in environment, ethnicity, socioeconomic status, and access to healthcare. All studies performed in Malaysia were conducted in Sabah. One study was a case report of an Australian traveler who went to Kalimantan, Indonesia, and was infected with *P. knowlesi* after spending months in the jungle [44] (see Figure 2). The types of research considered in our review are presented in Table 1. Of these studies, 50% relied on quantitative methods such as questionnaires and surveys to determine human behavior and activities contributing to exposure to *P. knowlesi* infection. 

Based on the analysis used, some studies provided descriptive demographic details [6,16,37,42,43,44]. Some studies performed a regression analysis with a *p* value of <0.05 to analyze the data gathered on human behavior, or activities that exposed them to *P. knowlesi* infection (see Supplementary Table S3). The respective odds ratio (OR) (95% CI) values of the behaviors and activities were not plotted due to the differences in the results and data collected, which could produce bias in this review. Owing to the complexity of the comparisons among the eligible studies, such as heterogeneous sample, different methods to measure behavior, and length of study, we decided not to proceed with a meta-analysis, to avoid incorrect conclusions [45].

### 3.2. Examination of Human Behavior in the Selected Studies

The ability to explore and understand complex linkages between human behavior and activities, land cover usage, and movement may help clarify the zoonotic disease risk to individuals and communities. While men are more likely to reside near plantations, the detection of *P. knowlesi* among women and children necessitates deeper exploration of their behavior and activities. The activities and behavior that exposed them to *P. knowlesi* infection have been quantified, but individual and community views, emotions, beliefs, and compliance of the community with antimalarial measures remain unclear. 

While the studies in this review provided diverse information on behavior, certain limitations remain in characterizing night-time activities, for example, the “what”, “when”, and the characterization of the human activities, such as routine household activities, livelihood activities, entertainment, outdoor sleeping, and traveling/visiting (Table 2). Some studies described behavior and activities using case reports and case series [42,44] (see Supplementary Table S3) and only one used observation to capture human (as well as mosquito) behavior [11]. Another study used a tracking device to monitor human movement and found that the median duration of movement was 16 days interquartile rate (IQR) (13.72–19.97) [38].

Only two studies incorporated both human and mosquito behavior [11,38]. One tracked the spatial direction of participants using the QStarz BT-QT13000XT GPS tracking device (QStarz, Taipei, Taiwan), programmed to continuously record coordinates at 1 min intervals for at least 14 days. The other was more robust, and mixed entomological studies exploring human movement through interviews and observations [11]. The researchers collected data on mosquito biting behavior and human movements at the same time and place. *Anopheles balabacensis* was the dominant species present around the participants houses, with more vectors outside the house. The vector was found to bite outdoors once it became dark [11]. 

### 3.3. Perceived Threats, Beliefs, and Misconceptions about P. knowlesi Malaria 

A qualitative study conducted among forest workers in Indonesia demonstrated a deeper understanding of human behavior by determining that belief, misconception, and lack of understanding regarding malaria risk might influence treatment-seeking behavior and noncompliance with antimalarial measures [40]. The poor perception of threat and awareness to *P. knowlesi* malaria could have been an issue in Sabang, Indonesia [40], and Surat Thani, Thailand [41]. The last reported *P. knowlesi* case in Surat Thani, Thailand, was in 2018 [41]. Forest workers reported low rates of preventive behavior as they perceived malaria as unthreatening, thus they did not adhere to antimalarial measures [40]. The forest workers’ believe that consuming certain foods or traditional herbs (*jamu*) or wearing charmed stones can protect them against malaria [40]. Other beliefs were that a chemical in bed nets could irritate the eyes and skin, and that bed nets caused breathing difficulty and led to hot and uncomfortable sleeping conditions [40]. Some also believed that *P. knowlesi* transmission occurred, but only in the forest rather than the surrounding community [40]. Supernatural beliefs about *P. knowlesi* being caused by evil spells in the forest also influenced workers’ treatment-seeking behavior, causing them to seek out traditional healers rather than medical doctors. Misconceptions about malaria, risk, and preventive behavior suggest a greater need for health education as some present malaria interventions are impractical [40].

### 3.4. Significance of Behavior and Activities to P. knowlesi Malaria Exposure

The behavior and activities that exposed communities to *P. knowlesi* infection were relatively similar across Malaysia, Thailand, Indonesia, and the Philippines. The activities included forest-related activities, such as traveling to the forest in Aceh Besar, Indonesia (adjusted odds ratio (AOR) 5.6, 95% CI 1.3–24.2, *p* = 0.020) [39], and in Sabah, Malaysia (AOR 2.48, 95% CI 1.45–4.23, *p* = 0.0010) [16]; and occupation-related activities like plantation work (AOR 3.50, 95 % CI, 1.34–9.15, *p* = 0.011) [16], farm or plantation work in Sabah, Malaysia (AOR 1.63, 95% CI 1.07–2.48, *p* = 0.025) [43], construction work [6], working in agricultural sectors (79%) [11], and similar exposure to forest workers in the Aceh province in Indonesia [40].

Activities like sleeping outdoors among the community studied in Sabah, Malaysia, showed a significant exposure to *P. knowlesi* malaria (AOR 3.61, 95% CI 1.48–8.85, *p* = 0.0049) [16]. This was similar to the community studies in Surat Thani, Thailand, where staying outdoors at night exposed people to mosquito bites in both January 2019 and May 2019 (AOR 3.15 and 2.26, 95 % CI 1.57–6.32 and 1.30–3.93, *p* = 0.001 and *p* = 0.004 [41]. Staying outdoors at night was described to be a significant risk factor for *P. knowlesi* malaria in Surat Thani, Thailand [41].

Other activities exposed individuals to *P. knowlesi* malaria, such as traveling to campsites and forest fringes for work in Sabang, Indonesia [6]. Not using a preventive measure at night in the forests, despite the presence of macaques, was described to be common [6,11,44]. In other parts in Indonesia, a study conducted in Aceh province described similar activities where traveling to work exposed one to *P. knowlesi* malaria; similarly, the practice of preventive measures against mosquito bites was poor [40]. In addition, some of the forest workers fed the macaques, caught them to sell, and brought them to their homes [40]. Similar conditions were described in Sabah, Malaysia, in which walking to the forest or plantation was associated with exposure to *P. knowlesi* malaria [37].

### 3.5. Demographic Factors

#### 3.5.1. Gender

There was clear heterogeneity between male and female behavior exposing them to *P. knowlesi* malaria. In Kota Marudu, Sabah, Malaysia, male gender is independently associated with increased risk for *P. knowlesi* infection (AOR 4.20, CI 95% 2.54–6.97) [16]. However, in Aceh province, Indonesia, gender was insignificant to the risk of *P. knowlesi* infection [39]. We also identified a sample bias as more men were recruited in the studies [11,16,40,41,42].

Serological evidence proved gender was not related to *P. knowlesi* infection, and both genders had a similar exposure to *P. knowlesi* based on their antibody response to *P. knowlesi* antigens [43]. A higher proportion of women were detected with asymptomatic infections [43]. In Thailand, people of both genders who performed forestry work were asymptomatic [41]. However, this finding may be biased owing to the sampling design and the small number of infected individuals. The study identified that both genders were exposed due to farm and forest work and clearing activities around the house. The spatial patterns and risk factors for *P. knowlesi* infection were different from other human malaria species, which highlights the need for *P. knowlesi*-specific disease control measures [43]. The serological results suggested infection occurred across age groups, and both genders, and present the possibility of a higher number of people exposed to *P. knowlesi* malaria, and from different demographic groups and geographical areas [43].

#### 3.5.2. Age

Children with no history of work or travel away from the house were also found to be infected with *P. knowlesi* in Sabah, Malaysia [16], and Indonesia [6,39]. It is believed that *P. knowlesi* transmission occurred in or around the house [6,11,16,39]. Thus, all age groups (and demographics) were at risk, as evidenced by serological investigation [43] of 10,100 individuals with a median age of 25 years (range 3 months to 105 years) and 2849 households in 180 villages; 5.1% (95% CI 4.8–5.4) were seropositive for *P knowlesi* malaria infection [43].

There was significant increased risk of *P. knowlesi* infection in those aged 15 years or older (adjusted odds ratio (AOR) 4.16, 95% CI 2.09–8.29, *p* < 0.0001) in Kota Marudu, Sabah, Malaysia [16], and among adults age 16–45 years as compared to <15 years old in Aceh, Indonesia, (AOR 14.0, 95 % CI 2.2–89.6) [39]. However, a population-based serological study from northern Sabah, Malaysia, and Palawan Island, detected asymptomatic *P. knowlesi* cases across all age groups and demographics [43].

#### 3.5.3. Place of Stay 

All of the study participants resided in villages, rural areas, and nearby forests. The majority of the population living in the village depended on core livelihood activities such as small-scale farming and plantation work [6,11,16,39,40]. Their places of stay—forested and hilly areas—are suitable for mosquito breeding, and in this study, *Anopheles* larvae were found near the homes [11]. 

### 3.6. Factors Related to Outdoor Activities 

Forest and agricultural activities are related to *P. knowlesi* malaria cases [6,11,14,37,38,39,41,42,46]. Six main forest activities in the deep forest and forest fringes expose forest workers to *P. knowlesi* malaria; specifically, cattle ranching, mining, logging, gathering rattan, forest patrolling, and agricultural activity [40]. More than 50% of patients admitted to the hospital in Sabah had slept overnight in the forest for the past four weeks, but a lower percentage (26.16%, *n*= 34/130 people) had slept overnight in the plantation [37]. In Aceh province, Indonesia, *P. knowlesi* malaria was detected in construction workers, contributing to a cluster of infections among workers [6]. In Sabah, Malaysia, farmers had a higher risk of contracting *P. knowlesi* malaria with an odds ratio of almost 2 compared with non-farmers (AOR 1.89, 95% CI 1.07–3.35, *p* = 0.028 [16]. Individuals who cleared vegetation were also more likely to be diagnosed with *P. knowlesi* infection with an odds ratio of almost 2 compared with those who did not (AOR 1.89, 95% CI 1.11–3.22, *p* = 0.020) [16]. However, peri-domestic transmission also occurred among individuals who did not perform forest-related activities [6,16]. 

A family cluster was found in a village in Sabang province, Indonesia, with no indication of engaging in forest-related activities or behaviors. The family house was located less than 500 m from the forest fringe, where macaques are commonly seen [6]. Moreover, people with a history of traveling to the forest were also exposed to *P. knowlesi* malaria [6,11,14,16,37,38,39,40,42,43,44] Almost 50% of *P. knowlesi* cases admitted to a tertiary center in Sabah worked within 20 min of the forest and plantation; however, the statistical significance was not measured [37]. 

While the majority of studies identified forests as factors that exposed the individual or communities to *P. knowlesi* infection, a study in Indonesia yielded contrasting results, with mostly statistically insignificant factors. Neither forest and non-forest work (*p* = 0.401) [39] nor overnighting in or near the forest were found to be significant [39]. However, visiting the forest for other reasons in the last month was found to be significant (AOR 5.62 95% CI 1.31–24.15, *p* = 0.02 [39]. The *Anopheles* mosquitoes were described as having adapted to deforestation caused by human activities. The mosquitoes start to bite outdoors immediately as darkness falls. The interaction of humans with mosquitoes and macaques increases the chance of zoonotic malaria transmission [6,14,16,40,42]. Ekawati et al. suggested improving malaria control and surveillance methods based on suitability to forest workers, by performing on-site, venue-based (VB), and peer-referral (PR) screening [40]. The malaria control program in Indonesia has begun discussion on appropriate surveillance and collaboration with the Ministry of Forestry and its Nature Conservation Unit on possible control strategies for zoonotic malaria [39]. The close interaction between humans, macaques, and mosquitoes increases the chance of being infected with *P. knowlesi*, and thus a new paradigm in managing this zoonotic malaria is needed [11]. 

The use of personal protective antimalarial measures, such as bed nets and repellent, appeared to be protective against mosquito bites in some studies. Only 45% of the positive *P. knowlesi* cases in a study conducted among patients admitted to the tertiary care center in Sabah, Malaysia, had used bed nets for protection [37]. Similarly, a cluster of *P. knowlesi* infection was described among the construction site workers in the Sabang province, Indonesia, who slept under temporary wood shelters for almost a month in the forest without using mosquito bed nets [6], even though there were common macaque sightings [6,40]. However, a study conducted in Aceh Besar, Indonesia, identified that sleeping under a bed net on the previous night was not significant to *P. knowlesi* malaria (AOR 2.75 95% CI 0.83–9.05, (*p* = 0.097)) [39]. Grigg et al. described that mosquito bed net use was not associated with protection against *P. knowlesi* infection, and did not seem to be protective against *P. knowlesi* acquisition due to the early peak biting times of *A. balabacensis* from 18:00 to 20:00 h, and that the mosquito mainly bites outdoors [16]. It is relevant to use conventional prevention activities among people who did not use a bed net during travel away from home [16].

The assessment of the usage of other self-protective measures, such as insect repellents, was uncommon. Only two studies assessed repellent usage against malaria exposure; in Aceh province, Indonesia [40], and in the travelers who had a long stay in Kalimantan, Indonesia [44]. Despite working in the forest, where macaques were commonly seen, he did not use any other personal vector measures such as mosquito nets and long clothing, nor did he receive malaria chemoprophylaxis. Similarly, the forest workers in Indonesia were reported to poorly practice malaria prevention measures, such as not using mosquito coils, repellent, medications, or bed nets [40]. 

### 3.7. Other Related Themes

Among the forest workers in the subdistrict in Aceh province, Indonesia, the primary health medical practitioner was perceived as unhelpful, not trustworthy, and disrespectful [40]. Complaints by people in the surrounding area regarding the services provided by the primary health center also influenced treatment-seeking behavior. Furthermore, the cost and the distance to receive treatment were barriers [40]. Many ignored their malaria symptoms and purchased medications from local pharmacies to continue working [40]. Among migrant workers, seeking treatment at a primary health clinic was problematic as they were not registered [40]. They also held misconceptions about the treatment received in the clinic and reported a fear of needles [40]. Additionally, traditional practices influenced individuals in Aceh, Indonesia, to seek treatment from traditional healers. Based on their tradition, they believed that a sick person was possessed by evil spirits, and traditional healers were perceived as more affordable, effective, and reliable [40]. 

The irregular use of bed nets was influenced by a lack of motivation for continuous usage. Participants described bed nets as uncomfortable as they made breathing difficult and made the sleeping environment hot [40]. They also felt that chemicals used on the net released an odor that irritated the eyes and skin. Some workers believed that malaria prevention was unnecessary as medications could be bought whenever illness occurred [40]. A lack of understanding of malaria risk may influence treatment-seeking behavior and suggested that health promotion and knowledge on preventive measures by these workers would be beneficial for the inhibition of the spread of malaria [40].

Finally, asymptomatic *P. knowlesi* infections were found in Surat Thani, Thailand, among adults engaged in agricultural activities [41]. In an exposed community in Sabah, Malaysia, and in the Philippines, asymptomatic malaria was found, showing varying levels of exposure to *P. knowlesi* malaria in all of the study sites [43]. Asymptomatic infection was believed to be caused by repeated exposure, resulting in the development of acquired immunity [41,43]. It is unclear how the low endemicity of the parasite can lead to development of immunity among individuals [43]. This evidence suggests the need for *P. knowlesi*-specific disease control measures, and that more people might be exposed to *P. knowlesi* than are identified at clinics; further, exposure to *P. knowlesi* might occur in different demographic groups and geographic areas than previously reported [43]. 

## 4. Discussion

This systematic review addresses the influence of human behavior and activities in relation to *P. knowlesi* malaria. It explores the methods used to measure and describe human behavior contributing to *P. knowlesi* malaria. Twelve eligible studies in this review revealed that human behavior and activities were related to exposure to *P. knowlesi* malaria. The identification is crucial in understanding how human behavior and activities increase contact between humans and mosquitoes to target more strategic vector control intervention across various settings.

Human behavior is complex and is influenced by multiple factors. While sociodemographic, environment, and outdoor activities were commonly described as significant factors to *P. knowlesi* infection, the contextual factors involving the characteristics of human behavior can be further explored to improve future *P. knowlesi* malaria programs. Our study highlights the influence of psychosocial factors such as belief, attitude, perceived threat, lack of motivation, and self-efficacy on individuals’ and communities’ malaria preventive behaviors. Other contributing factors were due to community perspective toward the infection and healthcare system. We argue that further exploratory studies should be performed in order to provide “logic” to the health outcome [46]. The understanding of human–vector contact patterns, and how they overlap in time and space, enables a more accurate representation of disease exposure [27,32]. Other factors such as social norms, perceived behavioral control, motivation to perform the behavior, and psychological factors such as attitude towards the preventive behavior and emotions, can play a role in malaria exposure [47,48,49]. Social context and behavioral factors are among the known attributes of community perceptions and behavioral practices on antimalarial preventive measures [29]. Beliefs concerning *P. knowlesi* malaria etiology influenced their attitude, self-efficacy, and ways of coping with antimalarial measures [40]. A proportion of the community in Indonesia had local supernatural beliefs towards malaria [40], and this perception was also present among the Orang Asli community in neighboring country, Malaysia [50]. This signals a gap in disease prevention as communities have different perspectives and beliefs towards malaria. The practice of sorcery, rituals, and remedies are believed to protect them from evil spirits and ghosts [40,50]. In addition, the availability of healthcare services alone is insufficient to ensure good health practices if individuals’ beliefs are unknown [40]. While most studies collected data by survey or questionnaire, analytical approaches should consider using exploratory study to detail the user perspective and experience in malaria exposure [27,29] to avoid participants responding to please the researcher [27]. Additional work is needed to address the remaining inquiries on human behavior and zoonotic malaria exposure.

The epidemiology of *P. knowlesi* infection is complex. While work and activities related to exposure play a role in malaria transmission in affected regions, other contributing factors, such as deforestation, disturbed the ecological balance of the area. As a result, these anthropogenic activities threaten the biodiversity and impact a higher risk of zoonotic malaria exposure as both monkeys and mosquitoes get closer to humans [2,6,11,12,40]. It is clear that *P. knowlesi* control is challenging, thus, individuals and communities need to culturally adapt to preventative measures, have sufficient knowledge about the issues, develop a positive attitude toward adopting health-supportive behaviors, gain support from and interact with others, and feel good about performing the behaviors [47,48,49]. Stakeholders must provide support to ensure a multi-collaborative effort in controlling this vector-borne disease [51], for example, by improving the housing structures, designing an innovative tool to avoid mosquito bites, and implementing the One Health approach [52]. 

Vulnerable communities living in rural and forested areas are at risk of *P. knowlesi* malaria. These communities have different norms and contexts that influence their living conditions. Malaria control strategies, especially those involving community education, should be tailored to fit each community depending on these factors [28,51]. Health promotion utilizing effective communication aids in preventive action should be considered, but such communication must be suited to the social context of the community [47,48,49]. A carefully designed malaria control program should consider the socio and behavior change (SBC) of the communities’ beliefs, drivers, facilitators, and motivation towards malaria preventive behavior. For example, if the objective is to encourage the wearing of protective clothing, the communication objectives should consider the individual’s emotions, social norms, and whether the tool is affordable [28]. Community-directed control programs may not be equally effective for all individuals in that community; however, and in this case, particularly competent individuals may act as advocates of SBC programs in the community [29,51]. Community participation in healthcare strategies should include all age groups to promote systematic, planned, and sustainable short- and long-term outcomes [51], as exposure to this zoonotic infection occurs in the young to elderly populations. Their participation can be facilitated by community dialogue and discussion of the social factors that may contribute to malaria exposure. This could provide collective opinions to help improve the social and structural environment, which in turn can be emphasized in malaria programs [29,51]. This method can help to provide community views to policymakers. Meanwhile, planning should include the application of behavioral change theories to guide researchers and policymakers to develop health and other behavior changes in targeted communities [34].

Standard preventive measures such as insecticide residual spraying (IRS) and bed nets are ineffective against *Anopheles* mosquito bites because the vectors are mainly outdoor biters [27,28,29]. The current interventions may have relatively little impact in controlling the zoonotic malaria [10,11,12]. While the success of human malaria control and elimination has been contributed proportionally by the usage of vector control measures such as ITNs and LLINs there are gaps in the degree of personal protective measures in zoonotic malaria infection. The integration of the SBC by building the motivation of individuals, improving their belief, attitude, perceived threat, and self-efficacy in avoiding mosquito bites, and increasing the utilization of healthcare services, can facilitate individuals’ protection against malaria [28]. Individuals’ attitudes and motivations can influence family members and communities. By complementing the understanding of disease transmission more effectively, the characteristics of human behavior in exposure could be targeted in future studies [27,32,33]. While time can be an issue for acceptance and behavior changes, cues for action can be continuously performed to promote consistent preventive habits and behaviors [28]. In return, such bottom-up approaches can help to sustain malaria intervention programs [28,29,51]. This is pertinent because voracious mosquitoes are a nuisance only in the presence of humans around them, thus suggesting the need for research to consider this interrelationship [29]. 

A paradigm shift in *P. knowlesi* malaria control measures is required [11,12]; with consideration of the problem from multiple angles and a creative, bottom-up approach, involving the community in its planning, implementation, and evaluation [28,29,51]. Intervention programs should respect local priorities and needs; such as acknowledging local social, economic, and political circumstances [28,29]. We must look at the negligible but real risk among vulnerable communities, to fulfil the United Nations Sustainable Development Goals (SDG) to end the epidemics of infectious diseases and neglected tropical diseases, including malaria, by 2030 [53]. To achieve the SDGs, the interruption of malaria transmission requires the integration of community involvement using communication within the communities at risk, strong political commitment, and continuous disease surveillance [53]. Programs should strive for greater community participation to strengthen disease control, ensure sustainability, and foster transformative SBC approaches [27]. The Malaria Policy Advisory Group (MPAG) concluded that the emergent epidemiological changes of *P. knowlesi* requires extensive research and continuous surveillance [3].

This systematic review will hopefully guide the structure and focus of future research concerning *P. knowlesi* malaria control programs. We argue that community awareness and knowledge alone is insufficient to assume a relationship to human behavior. It is imperative for policymakers to understand the context of the disease in that community and the various factors that drive and cause barriers to preventive actions [28,29]. Important next steps will be a standardized approach and further validation of data captured by different methodologies for collecting human behavioral data. A standardized approach will not only enable the assessment of human behavior but also help to track human and vector interactions. The gaps present in the usage of ITNs and other vector control interventions in the community could be described in greater detail. Once these exploratory study data have been gathered, a survey with uniform wording would allow for a more generalized study as well as evaluation of other control measures [47,54]. In addition, triangulation of different research methods to assess the factors contributing to persistent malaria transmission can contribute to a more in-depth understanding of disease transmission and consider communities’ feedback for the future improvement of malaria control strategies [54].

### Limitations

This review was limited by the heterogeneity of the studies explored. Additionally, the inclusion of only English language articles excludes findings from other languages. Moreover, only studies since 2010 were used because it was at this juncture that Malaysia started diagnosing *P. knowlesi* using specific PCR-based assays after a large focus of naturally acquired *P. knowlesi* zoonotic malaria infections were found in communities living in Kapit, Sarawak, Malaysia [1]. Moreover, present knowledge gaps exist regarding meteorological association/relations to *P. knowlesi* disease transmission, and how humidity, light, or moisture can increase or decrease the incidence/prevalence. The combination of methods and data collected shows that more studies need to be conducted to explore human behavior in relation to *P. knowlesi* malaria exposure.

## 5. Conclusions

We identified 12 studies that characterized how human behavior and activities exposed individuals or communities to *P. knowlesi* malaria; ranging from factors that influenced behavior such as disease misconception, beliefs, perceived threat, poor motivation, and self-efficacy towards using personal protective antimalarial measures, and others. Most studies used quantitative methods to capture human behavior related to *P. knowlesi* malaria. There has been a lack of exploratory studies aiming to understand human behavior exposing participants to *P. knowlesi* malaria, which provide the logic for the observed outcomes in a way that descriptive studies and surveys cannot explain the meaning in detail.

Further research in this area is needed, and it is hoped that the study findings may be used to develop future research and programs. The results suggest that for better and more effective malaria intervention policymakers should concentrate on understanding the details of human behavior and activities that might expose an individual or community to mosquito bites. A more socially acceptable intervention should be designed for the specific population while considering their social context, which can either become the driver of or barrier to malaria-preventive behavior. Public health control against *P. knowlesi* malaria is challenging, but future studies that do not overlook the importance of human behavior could provide hope in the form of novel information and interim strategies.

## Figures and Tables

**Table 1:** Inclusion and exclusion criteria for studies.

Inclusion Criteria	Exclusion Criteria
1. Present original (primary) data involving human behaviors, activities, or exposure to *P. knowlesi* malaria (e.g., bed nets usage, repellent usage, work exposure, and outdoor activities).	1. Articles that include mosquito biting rates or any entomological study without measuring human behavior.
2. Studies with sample populations from any malaria-endemic setting in Asia; studies involving travelers also accepted.	2. Laboratory studies, in vivo and in vitro, small communication reports, posters, and *Plasmodium* malaria studies not relating to *P. knowlesi.*
3. Quantitative or qualitative studies describing human behaviors during times when malaria transmission can occur (such as relationships, associations, or possible contributing factors); for example, presenting positive cases of *P. knowlesi* in the study site.	3. Reviews, protocols, guidelines, abstracts, preprints, conference papers, and commentary papers.
4. Published in English between January 2010 and December 2020.	
5. Published as a full peer-reviewed paper.	

**Table 2:** Studies including description of human activities at night.

Citation	Author	Location	The Night-Time Activities Identified in the Studies
[6]	Herdiana et al.	Indonesia	The subjects were exposed due to their livelihood activities, traveling history, and overnight sleeping. In case 2 (non-cluster cases), they had overnighted in Aceh but there was no description of the activities. In case 3 (non-cluster cases), they had overnighted at a campsite in Sabang due to work for 1 month. In case 8 (cluster cases), they spent the night at Sabang as the subject was an owner of a tourist shop
[11]	Manin et al.	Sabah, Malaysia	50% of the villagers would be indoors by 8 p.m. and out the next morning by 5 a.m. for work at the plantation
[14]	Fornace et al.	Sabah, Malaysia	No description on night-time behavior or activities
[16]	Grigg et al.	Sabah, Malaysia	The study subjects traveled overnight outside the village and slept outside their home
[37]	Barber et al.	Sabah, Malaysia	The study subjects overnighted in a forest for 4 weeks, overnighted in a plantation for 4 weeks, and also performed livelihood activities
[38]	Fornace et al.	Sabah, Malaysia	No description of night-time behavior or activities
[39]	Herdiana et al.	Indonesia	The study subjects were exposed due to their livelihood activities; they slept outside the house the previous night and had their workplace near or in the forest that required them to sleep overnight
[40]	Ekawati et al.	Indonesia	The study subjects were exposed due to their livelihood activities and occupation (agricultural work) and they returned home at night. These forest workers stayed overnight at a plantation (slept in a basic hut). Subjects who guarded the cattle at ranches were also at risk, including the police who patrolled for 24 h or training academy cadets and forest ranger patrols 1 and 2: Not described in detail, but a few nights to 2 weeks at a forest fringe plantation and up to 1 month at a plantation located deeper in the forest 3. Spent the night in two night shifts at the ranches 4. 24-h shift or 2–4 months of training academy 5. 2–3 h at night and 2 days per week or 8–12 days per month from 9 a.m. to 5 p.m
[41]	Shimizu et al.	Thailand	No description of night-time behavior or activities
[42]	Ngernna et al.	Thailand	No description of night-time behavior or activities
[43]	Fornace et al.	Sabah, Malaysia	No description on night-time behavior or activities
[44]	Figtree et al.	Australian traveler who went to Kalimantan, Indonesia	Night-time activities were not mentioned

## Data Availability

All data in this study were provided in the main manuscript and Supplementary Files.

## Supplementary Materials

The following are available online at https://www.mdpi.com/article/10.3390/ijerph19063675/s1: Supplementary Table S1, Summary of research databases and search strings used in this review; Supplementary Table S2, Quality analysis using the Joanna Briggs quality appraisal tools; Supplementary Table S3, Details of all eligible and included studies for review.
