# Supplementary material for: The Role of Human Behavior in Plasmodium knowlesi Malaria Infection: A Systematic Review
Source: Int J Environ Res Public Health. 2022 Mar 19;19(6):3675. doi: 10.3390/ijerph19063675 (PMC8953169; doi:10.3390/ijerph19063675)
Supplement: Supplementary file 1 [file ijerph-19-03675-s001.zip › ijerph-1603451-supplementary.pdf]

SUPPLEMENTARY MATERIAL

Supplementary Table S1 Summary of research databases and search strings used in this review

Supplementary Table S2 Quality analysis using the Joanna Briggs quality appraisal tools

Supplementary Table S3 Details of all eligible and included studies for review

Supplementary Table S1 Summary of research databases and search strings used in this review**.**

Database		Search strings	Advance setting
1	PubMed	((knowlesi) AND (behavior OR behaviour OR activit* OR exposure)) AND (human)) :.	95 studies published from 2010 to 2020
2	Web of Science	((knowlesi) AND (behavior OR behaviour OR activit* OR exposure)) AND (human))	61 studies published from 2010-2020
3	Science Direct	((Knowlesi) AND (behavior OR behaviour OR activity OR activities OR exposure) AND (human))	990 studies published from 2010 to 2020<br><br>After including only research articles and case reports, 390 results came back as search results
Total			546 studies<br><br>After duplicates were removed (n=38), the total studies that went through title and abstract screening were 508 studies

Supplementary Table S2 Quality analysis using the Joanna Briggs quality appraisal tools

No	Citation	Joanna Briggs Institute Critical Appraisal Checklist for Quality Analysis											
		**Cross sectional studies**	**Q1**	**Q2**	**Q3**	**Q4**	**Q5**	**Q6**	**Q7**	**Q8**			**Total**
1	14	Fornace et al.	Y	Y	Y	Y	N	N	Y	Y			6/8
3	38	Fornace et al.	Y	Y	Y	Y	N	N	Y	Y			6/8
4	39	Herdiana et al.	U	Y	Y	Y	U	N	Y	Y			5/8
5	41	Shimizu et al.	U	U	Y	Y	N	N	Y	Y			4/8
2	43	Fornace et al.	U	U	Y	Y	N	N	Y	Y			4/8
		**Case-control studies**	**Q1**	**Q2**	**Q3**	**Q4**	**Q5**	**Q6**	**Q7**	**Q8**	**Q9**	**Q10**	
6	11	Manin et al.	Y	Y	U	Y	Y	N	N	Y	U	Y	6/10
7	16	Grigg et al.	Y	Y	N	Y	Y	Y	Y	Y	U	Y	8/10
		**Experimental study**	**Q1**	**Q2**	**Q3**	**Q4**	**Q5**	**Q6**	**Q7**	**Q8**	**Q9**		
8	37	Barber et al.	Y	U	Y	U	Y	U	U	Y	Y		5/9
		**Case report**	**Q1**	**Q2**	**Q3**	**Q4**	**Q5**	**Q6**	**Q7**	**Q8**			
9	44	Figtree et al.	Y	U	Y	Y	Y	Y	N	Y			6/8
		**Qualitative studies**	**Q1**	**Q2**	**Q3**	**Q4**	**Q5**	**Q6**	**Q7**	**Q8**	**Q9**	**Q10**	
10	6	Herdiana et al.	U	Y	Y	U	N	Y	U	Y	Y	NA	5/10
11	40	Ekawati et al.	Y	Y	Y	N	Y	Y	Y	Y	Y	Y	9/10
		**Case series**	**Q1**	**Q2**	**Q3**	**Q4**	**Q5**	**Q6**	**Q7**	**Q8**	**Q9**	**Q10**	
12	42	Ngernna et al.	U	U	Y	U	U	Y	U	U	Y	NA	3/9

Q1 to Q10 are referring to the questions in the checklist, based on different type of study design by the Joanna Briggs checklist. Each type of study design has different sets of questions. Please refer to The Joanna Brigss website fo further details (https://jbi.global/critical-appraisal-tools).

The algorithm used in Supplementary Table S2 (Y-Yes; N-N; U-Unclear, NA-Not applicable

Supplementary Table S3 Details of all eligible and included studies for review

Citation	Citation (Country)	Title	Study design	Sampling method and sample size	The method used to capture human behavior	Data Analysis	Results	Study bias	Quality assessment
6	Herdiana et al. 2018 (Indonesia)	Two clusters of *Plasmodium knowlesi* cases in a malaria elimination area, Sabang Municipality, Aceh, Indonesia	Case series of two *P. knowlesi* clusters	Purposive sampling<br>--Cluster 1 (n = 8): Construction workers at a construction site on the far western tip of Sabang<br>--Cluster 2 (n = 3): A family (mother, two teenagers) from a residential location near the forest	A narrative review of the cases	Descriptive analysis	Factors that increased the risk for *P. knowlesi* infection<br>--Cluster 1: All cases were male construction workers with the same exposure risk and history of staying overnight in the forest, without antimalarial measures, and the macaques were common.<br>--Cluster 2: Possible transmission inside or close to the house. Thus, it caused an assumption of zoonotic or human-to-mosquito-to-human transmission of the parasite.	Not descrbed	Moderate
11	Manin et al. 2016 (Sabah, Malaysia)	Investigating the contribution of peri-domestic transmission to the risk of zoonotic malaria infection in humans	Entomology study with case-control study	Purposive sampling<br>--Cases were recruited from 180 *P. knowlesi* cases while a subgroup cases (n = 23) were recruited for entomology follow up in Kudat, Sabah<br><br>Random sampling<br>--A matched "control" household were recruited who shared similar environmental characteristics but negative for malaria.	1. Survey questionnaire<br>2. Interview<br>3. Observation<br>4.Entomology study	Descriptive analysis	Factors that increase the risk of malaria infection<br>1.Working at agricultural sector (78.6%; rubber estate, n = 13; oil palm plantation, n = 5; coconut plantation, n = 2; vegetable garden, n = 1)<br>2. Working in non-agricultural sector (14%; housewife, n = 1; others, n = 3)<br>3.Time to get to working place (54% in 10 minutes, 29% in 30 minutes).<br><br>Interview and observation<br>-- More than 50% of the villagers would be indoors by 8pm at night, and out by 5am on the next day to go to the plantations to work<br><br>Entomology study<br>--The proportion of human exposure to bites did not vary between case and control.<br>--Only 0.88% (13/1482) Anopheles was found to be infected with *P. knowlesi*, unable to make a conclusive prediction about infection risk.	Not described	Moderate
14	Fornace et al. 2019	Environmental risk factors and	An environmentally stratified,	Universal sampling	Survey questionnaire	Posterior estimation of odds ratios (OR)	Factors that increase the risk of *P. knowlesi* infection	1. Recall bias	High

	(Sabah, Malaysia)	exposure to the zoonotic malaria parasite *Plasmodium knowlesi* across northern Sabah, Malaysia: A population-based cross-sectional survey	population-based, cross-sectional survey	--Households in the Kudat, Kota Marudu, Pitas, and Ranau districts in northern Sabah, Malaysia (N = 10,100; *P. knowlesi* malaria cases = 3)		for fixed effects for *P. knowlesi* malaria infection exposure risk	1. Male sex (OR 1.245; 1.038–1.480)<br>2. Reported forest activities (OR 1.871; 1.447–2.368)<br>3. Reported contact with macaques (OR 1.871; 1.168-1.709)<br>4.Age, per 10 years (OR 1.332; 1.278-1.388)<br><br>Factor that has a protective effect against *P. knowlesi* malaria exposure<br>1. Use of insecticides (OR 0.765; 0.634–0.913)	2. Sample collected using filter paper may not have captured low-density parasite infection (asymptomatic cases)	
16	Grigg et al. 2017 (Malaysia)	Individual-level factors associated with the risk of acquiring human *Plasmodium knowlesi* malaria in Malaysia: A case-control study	Population-based, case-control study, 2-year period (December 5, 2012–January 30, 2015)	Purposive sampling:<br>– Cases (n = 414): microscopy positive, Polymerase Chain Reaction (PCR) confirmed malaria from 2 district hospitals (Kota Marudu and Kudat)<br>– Controls (n = 953): randomly selected 3 malaria negative community controls per case who were matched by village, within 2 weeks of case detection	Survey questionnaire<br>1. Demographics<br>2. Behavior<br>3. Residential malaria risk factors	Regression analysis	Factors that increased risk for *P. knowlesi* infection<br>1. Occupation:<br>– Farmers (aOR 1.89, CI 1.07–3.35, p = 0.028)<br>– Plantation work (aOR 3.50, CI 1.34–9.15, p = 0.011)<br>– Clearing vegetation (aOR 1.89, 95%CI 1.11–3.22, p = 0.020)<br>2. Sleeping outside the house (aOR 3.61, 1.48–8.85, p = 0.0049)<br>3. Travel (aOR 2.48, 1.45–4.23, p = 0.0010)<br>4. Being aware of the presence of monkeys in the past 4 weeks (aOR 3.35, 1.91–5.88, p < 0.0001)	1. Recall bias: P. k*nowlesi* patients who were aware of their diagnosis are more likely to recall seeing monkeys or describe childhood malaria episodes<br>2. Purposive sampling of symptomatic patients might exclude those who did not seek treatment, including asymptomatic cases<br>3. Variables' stepwise selection could exclude weaker associations with *P knowlesi* acquisition	High
37	Barber et al. 2012 (Malaysia)	A prospective comparative study of *P. knowlesi*, *P. falciparum,* and *P vivax* malaria in Sabah, Malaysia: High proportion with severe disease from *Plasmodium knowlesi* and *Plasmodium vivax* but no mortality with early referral and artesunate therapy	Integration of prospective, interventional and clinical-pathophysiological study	Purposive sampling<br>– All malaria (mono-infection) patients who were admitted to Queen Elizabeth Hospital in Kota Kinabalu, Sabah, Malaysia and diagnosed with *P. knowlesi* infection using PCR testing, from September 2010 to October 2011 (n=387)<br>– Study subjects were nonpregnant patients ≥ 12 years	A standardized data collection form that recorded the epidemiological, clinical, and baseline laboratory results of the patients	Descriptive analysis of the baseline demographic features of the *P. knowlesi* malaria cases	Factors that increase the risk of malaria infection<br>1.Occupation<br>– Farmer: n = 24 (18%)<br>– Plantation worker: n = 29 (22%)<br>2.Using mosquito nets: n = 59 (45%)<br>3.Forest exposure<br>– Live within 20 min walk of the forest: n = 71 (55%)<br>– Live within 20 min walk to plantation: n = 57 (48%)<br>– Work within 20 min walk of the forest: n = 64 (49%)<br>– Work within 20 min walk to plantation: n = 57 (44%)<br>– Overnight in the forest for past 4 weeks: n = 69 (65%)	1. Unable to assess all patients prior to commencing treatment<br>2. Reporting bias and no contact with true patients to explore more about their behavior as stated in the data collection form	Moderate

				old, and no major comorbidities<br>– They were recruited within 18 hours of commencing malaria treatment and had not previously been enrolled.<br>–From these subjects, 130 malaria patients had mix infection with *P. knowlesi* malaria			– Overnight in plantation for past 4 weeks: n = 44 (34%)<br>– >4 hours in forest for past 4 weeks: n = 87 (67%)<br>– >4 hours in plantation for past 4 weeks n = 44 (34%)<br><br>Factors that are not significant to *P. knowlesi* malaria infection<br>1.Farmer or plantation worker is not significant to *P. knowlesi* infection, with univariate analysis: 2.13 (0.97– 4.68), p = 0.059		
38	Fornace et al. 2019	Local human movement patterns and land use impact exposure to zoonotic malaria in Malaysian Borneo	Spatial-temporal ecology and entomology study: cross-sectional	Universal sampling<br>--Two rural communities in Northern Sabah, Malaysia, in Matunggong Kudat (n = 109) and Limbuak, Banggi Island (n = 134)	1. Remote-sensing data using QStarz BT- QT13000XT GPS tracking device<br>– Programed to record coordinates continuously at one-minute intervals for at least 14 days<br>– Trained fieldworkers visited the participant every two days to confirm device functioning or to replace batteries<br><br>2. Survey questionnaires<br>– Locations visited and GPS use	1. Descriptive analysis<br>2. Resource utilization functions, regression models with resource utilization as the response variable and using GPS count points to improve the model when there is missing data and location uncertainty (due to cloud cover of the GPS points)<br>3. Bayesian hierarchical spatiotemporal model<br>4. Individual exposure risk explored using a simple exposure assessment model where the number of infected bites received by an individual is the sum of bites by infected	Factors that increase the risk of malaria infection<br>1. Travelling around the study area. A maximum distance travelled from 0 km to 116 km (median: 1.8km) were identified from a total of 3,424,913 GPS points collected, which represented 6,319,885 person-minutes of sampling time.<br>2. The probability of an individual being in a location in space within a given time, varied by gender and occupation, termed by the utilization distributions (Uds).<br>**3.** Location and occupation: Individuals at the more rural Limbuak site covered the larger distances, and the largest distances were covered by individuals reporting primary occupations of fishing (n = 5) and office work (n = 9).<br>4. Substantial differences were reported between seasons in all movements (24 hr sampling).	Recall bias in questionnaire	High

						vector across all locations visited			
39	Herdiana et al. 2016 (Indonesia)	Malaria risk factor assessment using active and passive surveillance data from Aceh Besar, Indonesia, a low endemic, malaria elimination setting with *Plasmodium knowlesi*, *Plasmodium vivax*, and *Plasmodium falciparum*	Population-based, cross-sectional, over 19 months, from June 2014 to December 2015	Purposive sampling – Study subjects were recruited from passive case detection (PCD): 37 malaria cases from re-active case detection (RACD) of household members and neighbors residing within 500m radius of index case: 1495<br><br>*P. knowlesi* malaria cases (n = 20: 19 PCD, and 1 RACD)	Survey questionnaire on prevention and behavior including:<br>1. Travel<br>2. ITN ownership<br>3. Sleep under bed net the previous night<br>4. House sprayed in last 1 year<br>5. Slept outside the house the previous night<br>6. Workplace near/in forest<br>7. Visited forest in the last month for any reason	Descriptive statistic for *P. knowlesi* malaria cases	Factors that increased risk for *P. knowle*si infection<br>1. Visiting the forest in the last month for any reason (p=0.032)<br>--residence (n=5, 83.3%)<br>--work (n=5, 23.8%)<br>--other reason (n=3, 75%)<br><br>Factors that did not associate with risk to *P. knowlesi* infection:<br>1. Occupation category (p=0.112)<br>--non-forest related (n=9, 75%)<br>--forest-related job (n=11, 36.7%)<br>2. Travel history (p=0.071)<br>--no (n=12, 63.2%)<br>--yes (n=9, 37.5%)<br>3. ITN ownership (p=0.911)<br>--Yes (n=10, 50.0%)<br>--<1 ITN/2 people (n=7, 50%)<br>4. Slept under bed net the previous night (p = 0.264)<br>--no (n=11, 40.7%)<br>--yes(n=9, 56.3%)<br>5. Slept outside the house the previous night (p = n/a), everyone did not perform this behavior<br>6. Workplace near or in forest (p = 0.41)<br>--no (5, 62.5%)<br>--yes, required overnight (n=12, 38.7%)	1. Data collected using survey questionnaire method<br>2. Small sample size to justify the regression analysis	Moderate
40	Ekawati et al. 2020 (Aceh, Indonesia)	Defining malaria risks among forest workers in Aceh, Indonesia: A formative assessment	Qualitative study – five focus groups – 18 in-depth interviews with forest workers and key informants were conducted in each of four subdistricts in Aceh Besar and Aceh Jaya districts, from June to August 2016	Consecutive sampling<br>--historically passively detected index cases<br><br>Snowball sampling of referrals<br><br>Convenience sampling of community leaders	1. Focus group discussion<br>2. In-depth interview	Grounded theory	Factors that increase the risk of malaria infection (multivariate regression analysis**)**<br>1. Key informants described six main activities that occur in forested areas possibly can exposure forest workers to malaria infection: (i) agriculture; (ii) cattle ranching; (iii) logging; (iv) mining; (v) gathering rattan; and (vi) forest patrol<br>2. Farmers who cannot return home each evening reported spending a few nights to 2 weeks at forest fringe plantations and up to 1 month at plantations located deeper in the forest	1. No pilot test or member-checking was done<br>2. No training for researchers to conduct the study<br><br>Suggestion to avoid bias as described in the study<br>1. Mock interviews to gain experience<br>2. Refine the data collection guides and study procedures.	High

							to work and guard their crops against animals. During these periods, farmers sleep in simple huts.<br>3. Subjects reported low rates of prevention practice among forest workers.<br>4. Several subjects reported using bed nets, mosquito coils, repellent, and medication.<br>5. Not using mosquito nets or repellent at night<br>6. Working and sleeping outdoors at night<br>7. Only lighting a fire to ward off mosquitoes when sleeping in the forest		
41	Shimizu et al. 2020 (Surat Thani, Thailand)	Malaria cross-sectional surveys identified asymptomatic infections of *Plasmodium falciparum*, *Plasmodium vivax* and *Plasmodium knowlesi* in Surat Thani, a southern province of Thailand	Two cross-sectional surveys were conducted in January (dry season) and May (rainy season) of 2019 in areas of active transmission in the Surat Thani province of Thailand (18 villages in 4 districts)	Purposive sampling<br>--Male and female Thai nationals residing in the villages (n = 9418), aged ≥6 months were eligible, while individuals who were unable to communicate were excluded.<br>– January: n = 7034; May: n = 8671<br>– *P. knowlesi* malaria infection was detected in 3 individuals from 3 different villages of different districts	Survey questionnaire	Descriptive analysis and regression analysis	Factors that increase the risk of malaria infection<br>1. Staying outdoors during nighttime (AOR 3.15 and 2.26, 95%CI 1.57–6.32 and 1.30–3.93), p = 0.001 and p = 0.004, in January and May, respectively.<br><br>Factors that are not significant to malaria infection:<br>1. Bed net use<br>2. Insecticide residual spraying (IRS)	1. A male-biased risk, similar to elsewhere in Greater Mekong Subregion (Imwong et al. 2015a, Nguitragool et al. 2017, Zhao et al. 2018) was detected; presumably due to behaviors exposing males to more vector bites.	Moderate
42	Ngernna et al. 2019 (Southern border of Thailand)	Case report: case series of human *Plasmodium knowlesi* infection on the Southern border of Thailand.	Case series *of P. knowlesi* infection (detected from November 2017 until April 2018), from Songkhla and Narathiwat provinces of Southern Thailand (n=6)	Purposive sampling	Case report	Descriptive analysis	Factors that increases the risk of *P. knowlesi* malaria infection<br>1.Occupation (rubber tapper, oil palm plantation, herdsman, nontimber forest product finder, and wild animal hunter)<br>2.Activities (camping in the forest)<br>3.Monkey sightings at work site (forest)<br>4.Living in the rubber plantation<br>5. Frequent visit to forest near Malaysia-Thailand border, and present of wild monkeys habitats	Not described	Moderate

43	Fornace et al. 2018 (Malaysia and the Philippines)	Exposure and infection to *Plasmodium knowlesi* in case study communities in Northern Sabah, Malaysia and Palawan, The Philippines	Cross-sectional study	– Comprehensive sampling of all individuals residing within the study areas<br>– Those aged less than 3 months old, not primarily resided in the area for the past month, or could not be reached after three attempts were excluded<br>– 1. Limbuak, Banggi (n = 795)<br>2. Matunggong, Kudat (n = 11162)<br>3. Bacungan, Philippines (n = 546)<br>– Matunggong Kudat detected 4 *P. knowlesi* cases.<br>– Serology assessment<br>1. Limbuak: 93/795 – 11.7%<br>2. Matunggong: 79/1162 – 6.8%<br>3. Bacungan: 6/546 – 1.1%	Survey interview	Regression analysis	Factors that increase the risk of malaria infection<br>1.Age (p <0.001)<br>--under 15 years old (reference)<br>--15-45 years old (AOR 2.05 (1.30-3.22)<br>--45-60 years old (AOR 2.95 (1.70-5.11)<br>--over 60 years old (AOR 1.32 (1.32-4.58)<br>2. Main occupation (farm or plantation work) (p<0.0025<br>--No (reference)<br>-- Yes (AOR 1.62 (1.07–2.48)<br>2. Forest cover within 1 km (p=0.004)<br>--less than 30% (reference)<br>--over 30% forest cover (AOR 2.40 (1.29-4.46)	1. Non-randomized population sampling approach and limited geographical scale<br>2. Identifying environmental and population-level risk factors will require randomized sampling across a wider ecological gradient; community-level data on presence and absence of exposure and infection are required to understand spatial heterogeneity of disease transmission and develop and refine predictions of disease risk<br>3. The data on movement into different environments was not available for all survey participants	Moderate
44	Figtree et al. 2010 (Australia)	*Plasmodium knowlesi* in human, Indonesian Borneo	Case report	Purposive sampling<br>--One case of *P. knowlesi* infection in a traveller	Case report of the patient	Descriptive analysis	Factors that increase the risk of malaria infection<br>1.For the past 18 months, the patient had spent an average of 10 days per month working adjacent to a forest area in South Kalimantan Province, Indonesian Borneo.<br>2.Rainy season.<br>3. Subject did not use any personal vector avoidance measures (mosquito nets, long clothing, insect repellent)<br>4. Did not receive malaria chemoprophylaxis.	Not described	High
